# An Accurate and Convenient Method of Vehicle Spatiotemporal Distribution Recognition Based on Computer Vision

**DOI:** 10.3390/s22176437

**Published:** 2022-08-26

**Authors:** Zhiwei Chen, Yuliang Feng, Yao Zhang, Jiantao Liu, Cixiang Zhu, Awen Chen

**Affiliations:** 1 School of Architecture and Civil Engineering, Xiamen University, Xiamen 361005, China; 2 Fujian Key Laboratory of Digital Simulations for Coastal Civil Engineering, Department of Civil Engineering, Xiamen University, Xiamen 361005, China; 3 Xiamen Port Holding Group Co., Ltd., Xiamen 361012, China; 4 CCCC Second Harbor Engineering Co., Ltd., Wuhan 430040, China; 5 Xiamen Shuxin Construction Group Co., Ltd., Xiamen 361008, China

**Keywords:** vehicle spatiotemporal information, computer vision, pose estimation, field measurement

## Abstract

The Convenient and accurate identification of the traffic load of passing vehicles is of great significance to bridge health monitoring. The existing identification approaches often require prior environment knowledge to determine the location of the vehicle load, i.e., prior information of the road, which is inconvenient in practice and therefore limits its application. Moreover, camera disturbance usually reduces the measurement accuracy in case of long-term monitoring. In this study, a novel approach to identify the spatiotemporal information of passing vehicles is proposed based on computer vision. The position relationship between the camera and the passing vehicle is established, and then the location of the passing vehicle can be calculated by setting the camera shooting point as the origin. Since the angle information of the camera is pre-determined, the identification result is robust to camera disturbance. Lab-scale test and field measurement have been conducted to validate the reliability and accuracy of the proposed method.

## 1. Introduction

Traffic load is the main variable load on bridges. With the development of the transportation industry, traffic flow increases and it accelerates the fatigue cracking of bridge, which leads to the collapse of bridge. The information of traffic flow is important for bridge design, maintenance and reinforcement. Therefore, the accurate and effective identification of traffic load is crucial, including both weight and the spatiotemporal information of vehicle.

The weigh-in-motion (WIM) technique was proposed to obtain the weight of a passing vehicle [1,2], and it can measure both weight and number of axles. In many studies, data from WIM measurement was used to build the traffic load model and further investigate the effect of traffic load on the bridge. Caprani et al. [3] investigated the characteristic traffic load effects of mixing load on short and medium span bridges where data from WIM measured was used. O’Connor and O’Brien [4] compared various load effect extrapolation techniques using WIM data and analyzed the factors affecting the extrapolation accuracy. OBrien et al. [5] constructed a Monte Carlo simulation model by using WIM data to determine the traffic load effect on a bridge. Although the load effect can be estimated by using data of WIM measurement, it is still necessary to identify the transversal and longitudinal distribution of traffic load in real time, especially for bridge health monitoring [6]. Bridge weigh-in-motion (B-WIM) is different from the traditional WIM system [7]. Except for weight and number of axles, B-WIM can be arranged to obtain the load information. Scholars also used the measurement from the B-WIM system to estimate the vehicle location. Yuan et al. [8] analyzed the spatial distribution of vehicles on a bridge on the basis of previous studies. Yu et al. [9] proposed a novel method to identify the location information of vehicles by using the data from the B-WIM measurement. However, due to the characteristics of ill-conditioned equations, it is still difficult to identify multiple vehicles by the B-WIM algorithm. Hence, the existing WIMs alone cannot obtain the complete traffic load distribution information of the whole bridge. Other indirect methods to identify the location and amplitude of traffic load from structure response have also been investigated, and they can be classified into three categories: interpretive method [10], time domain method [11] and time–frequency domain method [12]. However, because these methods are based on beam theory, they can only be used to identify the longitudinal location of vehicle. In addition, they generally require a large amount of numerical calculations due to the usage of finite element (FE) model, which also limits their application.

In recent years, computer vision technology has been introduced in the field of structural health monitoring by detecting vehicles and identifying the location information [13,14,15]. Ojio et al. [16] proposed a contactless B-WIM system to weigh vehicles passing over a bridge without installing any sensors on the bridge. Feng et al. [17] introduced an innovative weighing method by visually estimating the contact pressure and contact area of vehicle tires. Later, various methods, such as background subtraction method [18], temporal difference method [19], Gaussian mixture model method [20], optical flow method [21], and match template method [22], were investigated to extract vehicle spatiotemporal information. Wang et al. [23] proposed a real-time robust algorithm to recognize targets in a complex background based on circle and line features, and they [24] further developed a high-precision target localization method for real-time visual measurement. Cao et al. [25] developed two algorithms to detect and track moving vehicles in aerial infrared image sequences. Jeong et al. [26] proposed an approach of spatiotemporal local-remote sensor fusion to identify vehicle location. Liu et al. [27] detected fast moving vehicles by using pose estimation with the Convex–Hull model. Lopez-Sastre et al. [28] improved the multi-vehicle tracking technique by using viewpoint estimation sensor. Tang et al. [29] developed an algorithm to extract the vehicle spatial distribution and 3D trajectory in a cross-camera traffic scene. However, these methods are highly sensitive to environmental conditions, which makes the vehicle spatiotemporal information acquisition less robust. With the development of deep learning technology, the powerful recognition ability of convolutional neural network (CNN) in image processing is recognized. Therefore, the identification of spatiotemporal information has become efficient and reliable. Some vehicle spatiotemporal information acquisition methods based on deep learning have appeared recently. Zhang et al. [30] proposed a method to obtain the spatiotemporal information of vehicles on the bridge based on DCNN technology and image calibration method. Zhou et al. [31] used the trained faster R-CNN model to detect the vehicle and employed Kalman filter to track its location. Gomaa et al. [32] developed a robust algorithm to detect vehicle by using CNN and optical flow. Jian et al. [33] developed a traffic sensing method that can automatically identify vehicle weight and speed. Xia et al. [34] proposed a traffic monitoring method for complex traffic scenes. Ge et al. [35] constructed a full bridge traffic load distribution monitoring framework based on YOLO-v3 machine vision. In fact, the spatial information of the vehicle can be obtained by using the dual target detection model, which can also detect the contour and tail of the vehicle and the visual principle. On this basis, Zhu et al. [36] used the YOLO-v4 detector to detect the vehicle and obtain the 3D boundary box.

Although deep learning technique makes the detection of multiple vehicles possible in complex scenes, there are still some problems in vehicle detection regarding vehicle spatial recognition. For example, the use of a camera to record vehicle motion needs to establish the relationship between the road surface coordinates and the image coordinates, where the prior spatial information of the road should be known. The common practice is to mark several points on the road and measure the distances between these points to establish the projection matrix on site. It can achieve satisfactory results. However, when the vehicle travels outside the area with the prior environment knowledge, it is difficult to estimate the location of vehicles. Hence, this method can only estimate the location of vehicle when it is in the area with prior environment knowledge. Jian et al. [33] developed a traffic sensing method that can automatically identify vehicle weight and speed. On the one hand, environmental vibration causes camera disturbance in long-term monitoring. The change in camera position also makes the identification results inaccurate, so the camera needs to be calibrated again, which is time-consuming. On the other hand, it usually difficult to obtain the environmental information of the whole bridge. Hence, it can hardly obtain the spatiotemporal information of vehicles on the whole bridge, which affects the assessment of bridge health condition.

In order to calculate the spatiotemporal information more conveniently, it is necessary to avoid prior work and reduce the interference induced by camera disturbance. In this study, an accurate and convenient method to identify the spatiotemporal information of passing vehicles is proposed based on computer vision. First, deep learning technology is used to detect vehicles in the video. Then, camera calibration is conducted to obtain the camera internal parameter matrix and distortion coefficients. Finally, the camera pose is used to estimate the spatial relationship between the camera and the vehicle to obtain the vehicle spatiotemporal information.

The main contributions of this study are summarized as follows. (1) Prior environment knowledge can be avoided so that it is more convenient in practice. (2) The adverse effect of camera interference can be reduced. (3) The feasibility of YOLO-v5 on identifying passing vehicles was investigated and discussed.

The following text is organized as: Section 2 briefs the framework of the proposed method. Section 3 introduces the YOLO-v5 detector framework, explains the generation process of the data set, and provides the performance analysis of the detection model. Section 4 presents the proposed method to identify vehicle spatiotemporal distribution. In Section 5, the accuracy of spatiotemporal information recognition and robustness to camera disturbance are verified by lab tests. Section 6 shows the field measurement by using the proposed method, and conclusions and discussions are summarized in Section 7.

## 2. Framework of the Proposed Method

**Figure 1:**
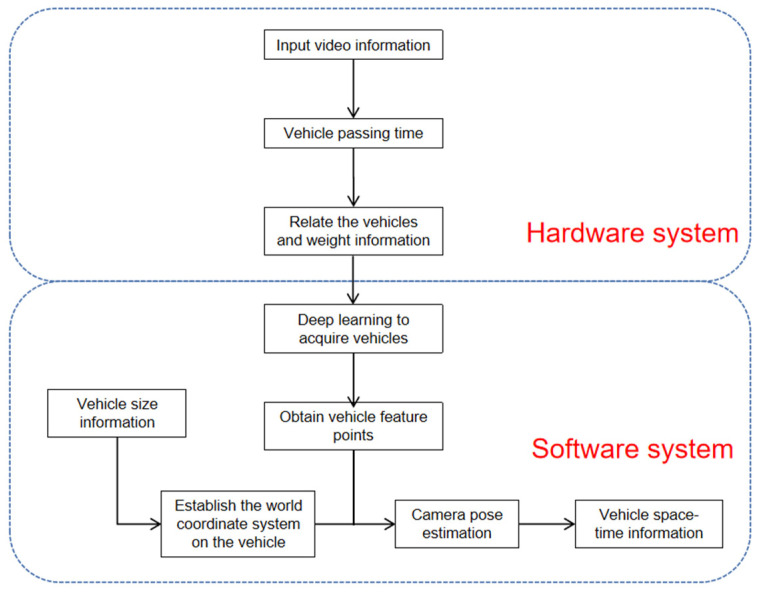
Framework of the proposed method.

**Figure 2:**
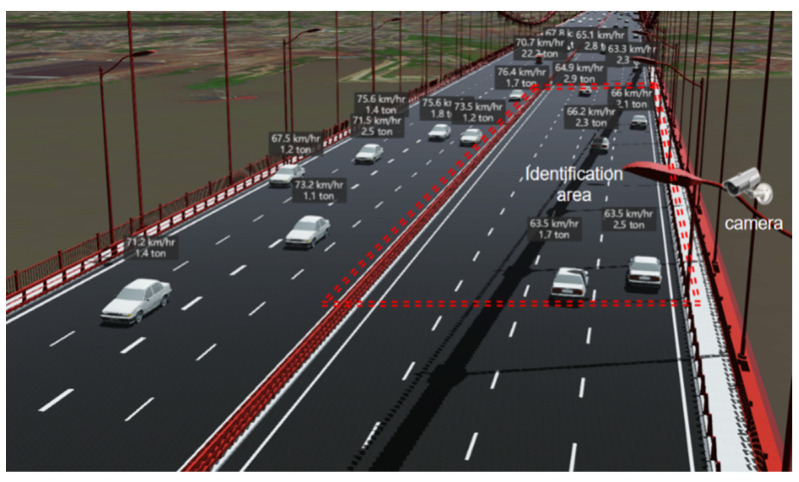
On-site hardware layout.

The framework of the proposed method consists of a hardware system and software system, as shown in Figure 1. The hardware system includes surveillance cameras to record video of traffic flow (Figure 2) and the software system includes a dynamic vehicle detection module and vehicle tracking module to identify the spatiotemporal information of passing vehicles.

The main task of the dynamic vehicle detection module is to identify vehicles in the video. Although a passing vehicle can be easily detected by background subtraction and Gaussian mixture model method, it is vulnerable to weak light and its performance on detecting multiple vehicles is poor. Deep learning-based target detection methods such as R-CCN and Fast R-CNN usually have two steps, which detects vehicles in real time with difficulty. Therefore, YOLO-v5 was adopted in this study.

The main task of the vehicle tracking module is to calculate the location of the identified vehicle and reconstruct its trajectory. Most of the existing methods need prior environment knowledge, which is not convenient to obtain in practice. Therefore, a novel vehicle tracking method is proposed, which can identify the position information of the vehicle without prior environment knowledge. It mainly uses the principle of pose estimation to obtain several marked points from the vehicle when the vehicle size is known and uses the position information of the marked points to calculate the shooting position of the camera. Because the marked points move with the vehicle, the relative position relationship between vehicle and camera at each time should be calculated. The camera position remains unchanged, and the mileage information of the vehicle can be deduced accordingly. This method does not need prior environment knowledge, overcomes the influence of camera disturbance, and is suitable for large-scale and long-term monitoring.

## 3. Vehicle Detection Based on YOLO-v5

### 3.1. YOLO-v5 Model

Several versions have been released since the start of the YOLO detector. Some scholars have achieved good recognition results by using YOLO-v3 and YOLO-v4. The network structure of YOLO-v3 is a classic one stage structure, consisting of four modules: Input, Backbone, Neck and Prediction. YOLO-v4 has involved many innovations on the basis of YOLO-v3. From YOLO-v4 to YOLO-v5, great improvements have been made. The size of YOLO-v5 is nearly 90% smaller than that of YOLO-v4 and the accuracy of YOLO-v5 is equivalent to that of YOLO-v4. In the official code of YOLO-v5, there are four versions in the target detection network, namely, YOLO-v5s, YOLO-v5m, YOLO-v5l and YOLO-v5x. The accuracy and computational efficiency of the four versions are slightly different. YOLO-v5 not only keep the advantages of high precision and high efficiency of previous products, but also provides users more options. Moreover, considering the follow-up long-term monitoring, it is necessary to implement a small size target detection model in embedded devices. Therefore, YOLO-v5 is used as the detector in this study.

**Figure 3:**
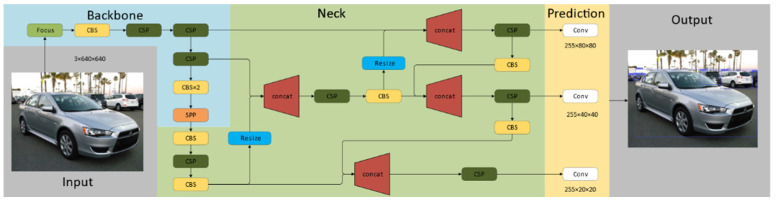
YOLO-v5s network structure.

Figure 3 shows the network structure of YOLO-v5s.The network is mainly divided into four parts: Input, Backbone, Neck and Prediction. The differences between YOLO-v5 and other versions are briefly introduced. In the Input module, the function of self-adaptive image scaling is improved, which scales the original image to a standard size and sends it into the detection network. YOLO-v5 modifies the letterbox function and adaptively adds the least black edges to the original image to improve speed. In the Backbone module, the Focus structure is added, in which the slicing operation to slice the input image into a feature map is the most important. For a 640 × 640 × 3 image, it first becomes a 320 × 320 × 12 feature map, and then transforms to a 320 × 320 × 32 feature map after a convolution operation. YOLO-v5 has two CSP structures, CSP1_X structure is applied to the Backbone, and CSP2_X structure is applied to the Neck. The Neck adopts FPN+PAN structure, and the size of the image after feature extraction becomes smaller. Through three times of up-sampling, the image is mapped from small resolution to large resolution, and three feature maps with different sizes are obtained for the subsequent prediction. Finally, the loss function is expressed as:

(1) $$\mathrm{CIOU}\_\mathrm{Loss}=1-(\mathrm{IOU}-\frac{\mathrm{Distance}\_2^{2}}{\mathrm{Distance}\_C^{2}}-\frac{v^{2}}{\left(1-\mathrm{IOU}\right)+v})$$

(2) $$v=\frac{4}{\pi^{2}}(\arctan\frac{w^{gt}}{h^{gt}}-\arctan\frac{w^{p}}{h^{p}}{)}^{2}$$

where v is the parameter to measure the consistency of the aspect ratio.

### 3.2. Model Training and Performance Analysis

**Figure 4:**
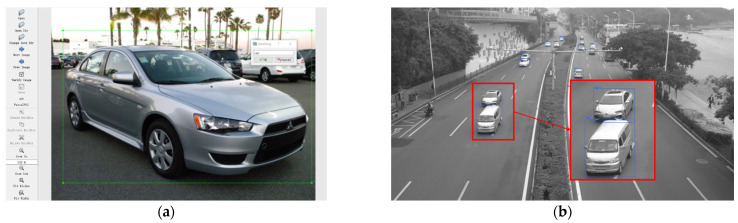
(**a**) Label the vehicle using the data labeling tool. (**b**) Vehicle detection by YOLO-v5.

The model training needs a vehicle dataset. The vehicle images in the dataset in this study were either pictured by camera or downloaded from the internet. The labeling tool was used to label the vehicle in the figure, as shown in Figure 4a. The dataset was divided into two parts by random sampling: one was the training set including 80% of the images and the other was the test set including 20% of the images. The former was used to train the network and the latter was used to evaluate the detector.

The actual traffic flow video was used to verify the vehicle recognition capability of the model in the field measurement. Figure 4b shows the vehicle detection results at 10 am. The position information and category probability in the image are provided in the form of a bounding box. It was found that the model used in this study can accurately detect the vehicle under the condition of fast driving, and it has good detection results when multiple targets are presence in the vision. However, when the vehicle has obvious occlusion, vehicle contour positioning may lead to large errors.

## 4. Vehicle Spatiotemporal Distribution Recognition

### 4.1. Corner Points Marking

Four points on the passing vehicle are marked first, which are used to identify the location of vehicle. Generally, the marked points should be on geometric corners of the vehicle. This is because the captured vehicle boundary or geometric corner has significant color change and brightness change, which is helpful to detect the marked points in the image and obtain the corresponding 2D coordinates. For any image in the video, once the vehicle is identified, the marked points can be detected and their 2D coordinates can be obtained accordingly. When there are multiple vehicles in the image, all vehicles can be identified simultaneously by YOLO-v5, and the marked points on each vehicle can be detected separately.

### 4.2. Determination of the Relative Position of the Camera and Vehicle

A method to obtain the relative position of camera and vehicle by using the marked point information in world coordinates is proposed in this study. In fact, the number of marked points, n, is important in this method.

**Figure 5:**
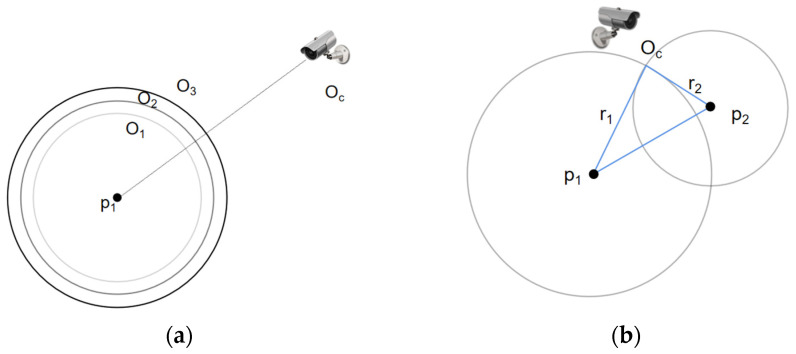
(**a**) Relative position of the camera and vehicle (n=1). (**b**) Relative position of the camera and vehicle (n=2 ).

When there is only one marked point (n=1) on the vehicle, as shown in Figure 5a, P_1_ is the marked point and O_C_ is the optical center of the camera. Suppose that the marked point is in the center of the image; then, P_1_-O_C_ is the Z-axis of the camera. Then, the camera may be at any point on a sphere with an arbitrary radius and center of P_1_; hence, there are infinite solutions and the relative position of camera and vehicle cannot be determined.

When there are two marked points (n=2) on the vehicle, as shown in Figure 5b, namely, P_1_ and P_2_, the additional constraint condition makes O_C_-P_1_-P_2_ form a triangle. Since the positions of P_1_ and P_2_ are determined, the edge P_1_P_2_ of the triangle can be determined. In addition, the vector O_C_P_1_ and the direction angle of O_C_-P_2_ can also be determined accordingly. Therefore, the length of O_C_P_1_, r_1_, and the length of O_C_P_2_, r_2_, can be calculated. In this case, two spheres are obtained: one has center P_1_ and radius r_1_ and the other has center P_2_ and radius r_2_. Obviously, the camera is located at the intersection line of the two spheres, so there are still infinite solutions.

When there are three marked points (n=3) on the vehicle, that is, there is one more marked point, P_3_, there should be one more sphere with center P_3_ and radius O_C_P_3_. The camera is located at the intersection of the three spheres. Since there are four solutions, one may need additional information to determine the solution.

**Figure 6:**
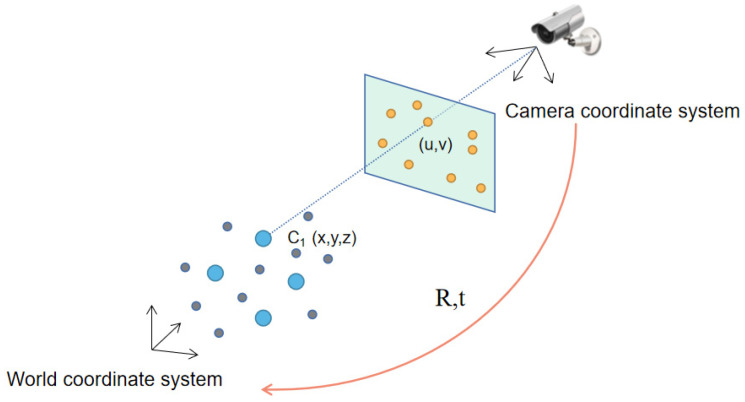
Relationship between 3D points (world coordinates) and 2D points (image coordinates).

When there are more than three marked points (n>3) on the vehicle, all spheres should have only one intersection point, and it is the exact location of the camera. Hence, at least four marked points should be selected on a vehicle. Perspective-n-point (P-n-P) technique is usually adopted to match point on 3D object to 2D point. The relationship between the coordinates of point on the 3D object in the world coordinates and coordinates of corresponding point in the image plane can be constructed, and the pose of camera (six degrees of freedom: position coordinates and three direction angles) can be determined accordingly. As shown in Figure 6, when the coordinates of point C_1_ in the world coordinate system and the coordinates of the corresponding point in the image plane are known, combined with the internal parameter matrix and distortion coefficient of the camera, the rotation vector and translation vector transformed from the world coordinate system to the image plane coordinate system can be calculated.

If the camera position remains unchanged and the world coordinate system is established on the moving object, the displacement information of the moving object can be obtained. It should be noted that the coordinates of marked point in world coordinate system can be determined either through the depth map or by setting the world coordinate system during initialization. Therefore, the pose estimation method does not need a polar constraint and can obtain better motion estimation. As a summary, the relationship from the world coordinate system to the pixel coordinate system has been proposed by predecessors [37].

Before actual measurement, the internal parameter matrix and distortion coefficient of the camera should be calibrated. A chessboard composed of black-and-white square intervals was used as the calibration object for camera calibration, and the orientation of the chessboard was changed many times to capture images. After calibration, the parameters and distortion coefficient were obtained for actual measurement.

### 4.3. Identification Vehicle Spatial Information by Using Pose Estimation

The Direct Linear Transformation (DLT) method was used to determine the external parameters of the camera. For a specific point $P_{w}$ having coordinates (X,Y,Z) in the world coordinate system, its corresponding pixel coordinate (x,y) can be expressed as:

(3) $$\left[\begin{array}{c} x\\ y\\ 1 \end{array}\right]=KT\left[\begin{array}{c} \begin{array}{c} X\\ Y \end{array}\\ \begin{array}{c} Z\\ 1 \end{array} \end{array}\right]$$

where K is the internal parameter matrix of camera and T is the transformation matrix. K is predetermined in the calibration, while T is to be determined.

Multiplying $K^{-1}$ on both sides of Equation (1), one can obtain:

(4) $$K^{-1}\left[\begin{array}{c} x\\ y\\ 1 \end{array}\right]=\left[\begin{array}{cc} R & t \end{array}\right]\left[\begin{array}{c} \begin{array}{c} X\\ Y \end{array}\\ \begin{array}{c} Z\\ 1 \end{array} \end{array}\right]=\left[\begin{array}{cccc} r_{11} & r_{12} & r_{13} & t_{1}\\ r_{21} & r_{22} & r_{23} & t_{2}\\ r_{31} & r_{32} & r_{33} & t_{3} \end{array}\right]\left[\begin{array}{c} \begin{array}{c} X\\ Y \end{array}\\ \begin{array}{c} Z\\ 1 \end{array} \end{array}\right]$$

where R is the rotation matrix and t is the translation vector. Therefore, once the transformation matrix is obtained, the coordinates (X,Y,Z) in the world coordinate system can be calculated by using Equation (2). For one pair of marking points, there can be two equations. The transformation matrix has 12 elements, so it needs at least 6 pair of marking points to obtain the solution. However, since R is the rotation matrix, it is an orthogonal matrix with determinant 1 and there are constraints that $RR^{T}=1$ and det(R)=1. Hence, the following equations can be obtained:

(5) $$\{\begin{array}{c} \begin{array}{c} r_{11}{}^{2}+r_{12}{}^{2}+r_{13}{}^{2}=1\\ r_{11}r_{21}+r_{12}r_{22}+r_{13}r_{23}=0 \end{array}\\ \begin{array}{c} r_{11}r_{31}+r_{12}r_{32}+r_{13}r_{33}=0\\ r_{21}{}^{2}+r_{22}{}^{2}+r_{23}{}^{2}=1 \end{array}\\ \begin{array}{c} r_{21}r_{31}+r_{22}r_{32}+r_{23}r_{33}=0\\ r_{31}{}^{2}+r_{32}{}^{2}+r_{33}{}^{2}=1 \end{array} \end{array}$$

Therefore, the transformation matrix can be obtained by three pairs of marking points. In order to improve the accuracy, four pairs of marking points were selected in this study and the transformation matrix was solved by using least squares method. The pose minimizing the reprojection error can be found by using Levenberg–Marquardt optimization. Then, the rotation angle of each axis of the camera $\left(\theta_{cx},\theta_{cy},\theta_{cz}\right)$ in the world coordinate system can be calculated by using the rotation matrix R. After obtaining the three rotation angles of the camera, the coordinates of the camera $\left(x_{c},y_{c},z_{c}\right)$ in the world coordinate system can be determined as well. Finally, as long as the exact location information of the camera in the world coordinate system is known, including both rotation angles and coordinates, the coordinates of point on a 3D object in the world coordinate system can be calculated using Equation (1) and the motion information or trajectory of the point can be obtained.

**Figure 7:**
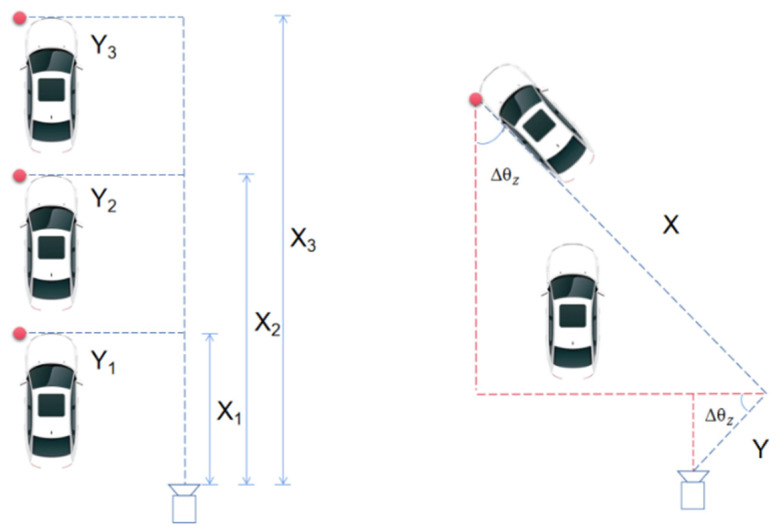
Determination of vehicle location in straight and curved paths.

When the vehicle travels along a straight line, the spatiotemporal information of the vehicle can be obtained by directly recording the coordinates of X and Y in the world coordinate system at each time, as shown in Figure 7. When the vehicle travels along a curved path, the world coordinate system changes with the vehicle driving direction. In this case, the directly obtained coordinates X and Y cannot correctly reflect the vehicle location information. The coordinates should be corrected by using the turning angle of the vehicle. Because the camera is fixed during the measurement, the *Z* axis in the world coordinate system is perpendicular to the ground, so $\theta_{cz}$ can reflect the turning angle of the vehicle, which is also called the yaw angle. The yaw angle of the vehicle before and after entering the curve is recorded as $∆\theta_{z}$. By using the geometric relationship, the vehicle location information $X_{t}$ and $Y_{t}$ are obtained as:

(6) $$X_{t}=X\cos∆\theta_{z}+Y\sin∆\theta_{z}$$

(7) $$Y_{t}=X\sin∆\theta_{z}-Y\cos∆\theta_{z}$$

If the vehicle is on the right side of the camera and turns to the right, the location information can be calculated by Equations (6) and (7). However, when the vehicle on the left side of the camera turns to the right and the vehicle on the right side of the camera turns to the left, Equations (6) and (7) can be transformed as:

(8) $$X_{t}=X\cos∆\theta_{z}+Y\sin∆\theta_{z}$$

(9) $$Y_{t}=Y\cos∆\theta_{z}-X\sin∆\theta_{z}$$

**Figure 8:**
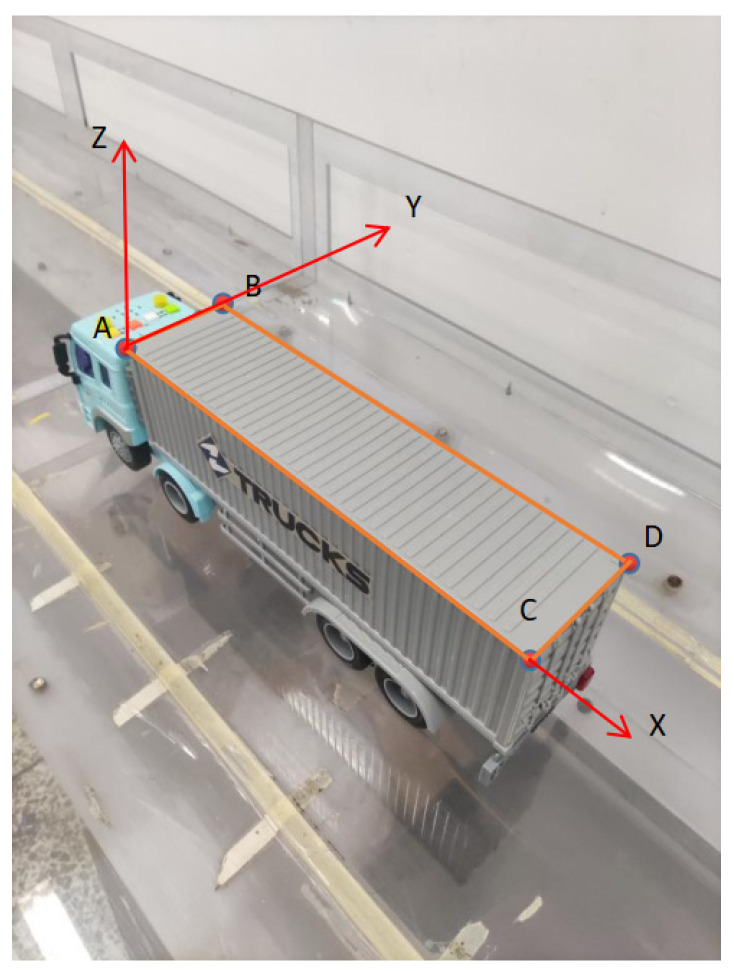
The selected four points and world coordinate system on the truck.

In practice, a vehicle usually has a clear boundary and the size information can be easily obtained. Hence, the world coordinate system is established on the vehicle, and the location information of four points is selected to calculate the transformation matrix. The four points can be selected from a surface of the vehicle. Figure 8 shows an example: Four points, A, B, C, and D on the top surface are selected and the world coordinate system is established on the truck. The pixel coordinates of A, B, C and D are tracked and matched with the world coordinates to obtain the rotation matrix and translation vector of different planes of the vehicle during moving. Since the camera is fixed, therefore, the six degrees of freedom of the vehicle can be deduced by using Equations (4) and (5).

### 4.4. Summary of the Proposed Method

The steps of the proposed method are summarized as follows:

Step 1: The internal parameter matrix and distortion coefficients of camera are obtained through camera calibration.

Step 2: At least 4 pairs of marked points are selected and their coordinates in world coordinate system and pixel coordinate system are determined.

Step 3: The external parameter matrix of the camera is calculated by using DLT method.

Step 4: The six degrees of freedom of the camera are obtained according to the external parameter matrix.

Step 5: The location information of the vehicle in the world coordinates is deduced from the video recorded by the camera.

## 5. Verification by Lab-Scale Tests

**Figure 9:**
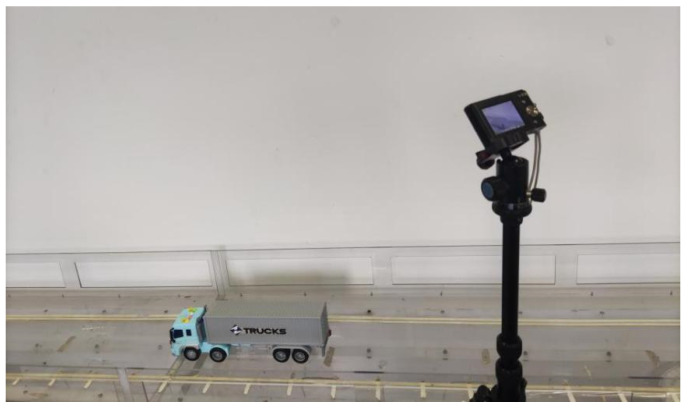
Layout of the model truck and camera.

In order to verify the proposed method and investigate the influence of camera disturbance on the proposed method, a total of four tests were conducted in the lab where a model truck was used. Figure 9 shows the layout of the model truck and camera in the lab. In Test 1 to Test 4, the model truck moved along the straight line on the model continuous bridge.

### 5.1. Verification of the Accuracy of the Recognized Vehicle Spatiotemporal Information

Test 1 and Test 2 were conducted to verify the accuracy of the proposed method. In order to obtain the accurate spatiotemporal information of the vehicle, several points on the bridge deck were marked, which were uniformly arranged along the straight line. Hence, the locations of these points on the bridge deck were known. When the vehicle moved along the bridge deck, the vehicle was recorded in the form of images, and its location was estimated by the proposed method, which was further compared to the results obtained by the marked points to obtain relative error. In Test 1, the camera was placed at one location near the track, as shown in Figure 9. In Test 2, the location of the camera changed, and the time history of location information of the truck was identified again to investigate the influence of camera location on identification accuracy.

**Figure 10:**
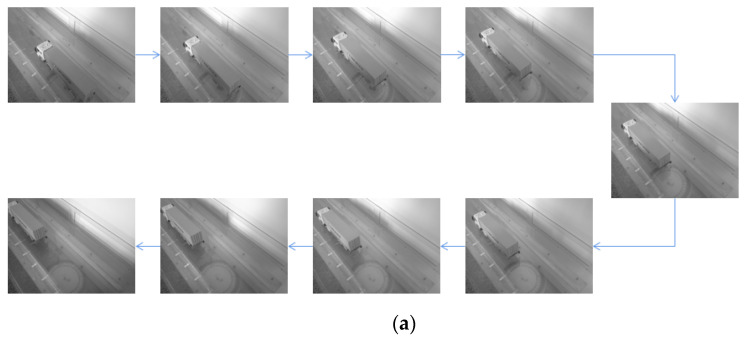

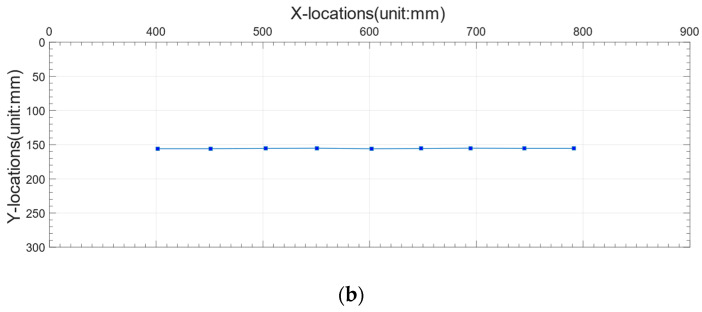
(**a**) The model truck photographed by the camera in Test 1. (**b**) Trajectory of the model truck in Test 1.

Figure 10a shows the images of the model truck taken by the camera in Test 1. The location of model truck in each image was identified by the proposed method and the spatiotemporal information is summarized in Table 1. The first eight columns show the pixel coordinates of the selected four points. The following two columns show the identified coordinates of the center of rectangle ABDC in world coordinate system, and the last column shows the percentage error of the Y coordinate in world coordinate system. It is observed that the maximum relative error is only 1.07%, indicating that the proposed method can identify the spatiotemporal information of passing vehicles with high accuracy. Figure 10b shows the trajectory of the model truck in Test 1.

**Figure 11:**
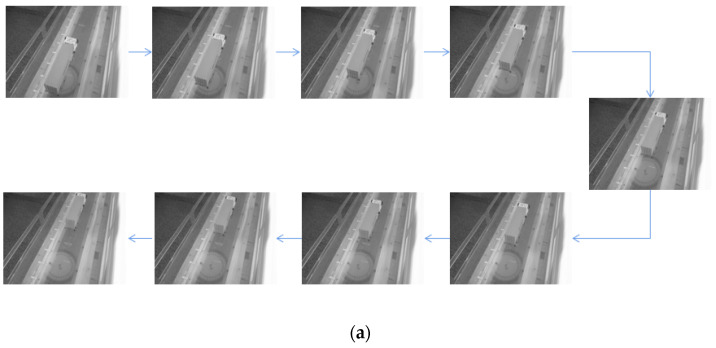

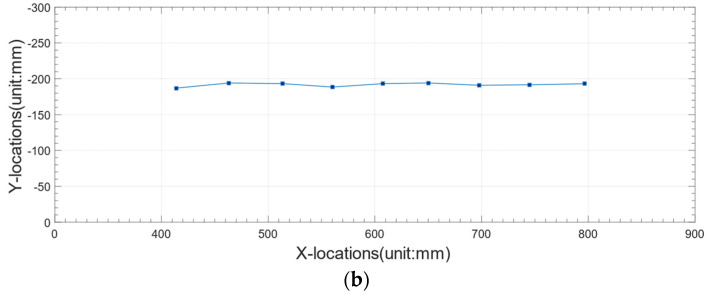
(**a**)The model truck photographed by the camera in Test 2. (**b**)Trajectory of model truck in Test 2.

Figure 11a shows the images of the model truck taken by the camera in Test 2, in which the camera was moved to the other side of the vehicle. The location of model truck in each image was identified and the spatiotemporal information is summarized in Table 2. The maximum relative error is 3.49%, also indicating that the proposed method can accurately identify the spatiotemporal information of passing vehicles even if the location of camera changes. Figure 11b shows the trajectory of the model truck in Test 2.

Test 1 and Test 2 prove that using the proposed method to calculate the vehicle spatiotemporal information has high accuracy, and it is not limited by the camera installation location. Hence, the camera can be installed in a specific location on the actual traffic road, which is convenient in practice.

### 5.2. Investigation of Robustness to Camera Disturbance

**Figure 12:**
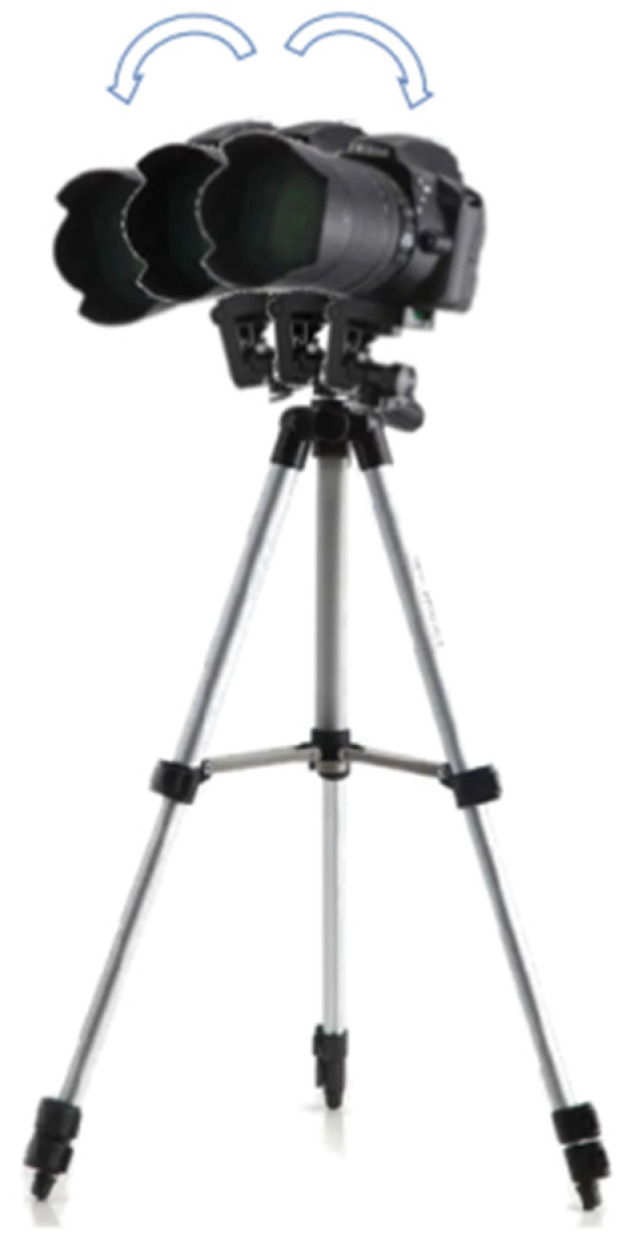
Illustration of camera lens disturbance.

**Figure 13:**
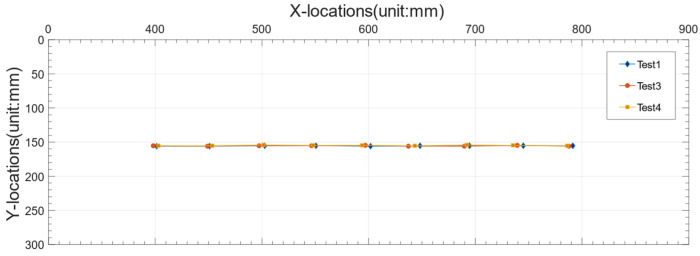
Trajectories of the model truck in Test 1, Test 3 and Test 4.

Test 3 and Test 4 were conducted to investigate the robustness of the proposed method to camera disturbance. The location of camera was kept the same with that in Test 1, but the camera was rotated randomly, as shown in Figure 12. In the three tests, the model truck moved along the same path, but the speed had slight variations. Figure 13 presents the trajectories of the model truck in Test 1, Test 3 and Test 4, and it may be concluded that the proposed method is robust to camera disturbance, which is beneficial in real applications.

## 6. Verification by Field Measurement

**Figure 14:**
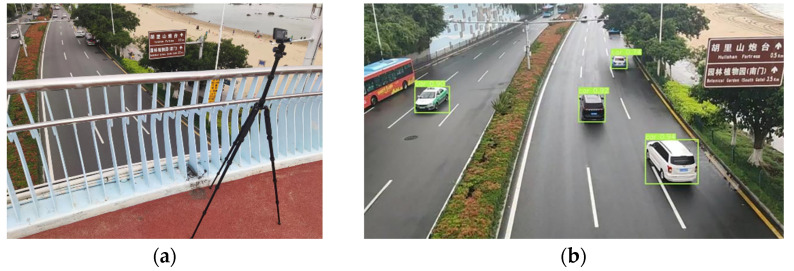
(**a**) Camera on pedestrian overpass. (**b**)Detection of vehicles in field measurement.

Field measurement has also been carried out to further validate the proposed method. The camera was set on a pedestrian overpass, as shown in Figure 14a. The measurement was conducted in the morning when the traffic condition was good and there was no environmental interference. The locations of vehicles on the three lanes on the right were identified.

The camera was calibrated before measurement. The vehicles were first detected by YOLO-v5s, as shown in Figure 14b. Four points of the shadow area at the bottom of the vehicle were selected as the marked points to establish the world coordinate system. Although one point was blocked by the vehicle itself, the pixel coordinates of the blocked points can be approximated because the bottom is a regular rectangle.

**Figure 15:**
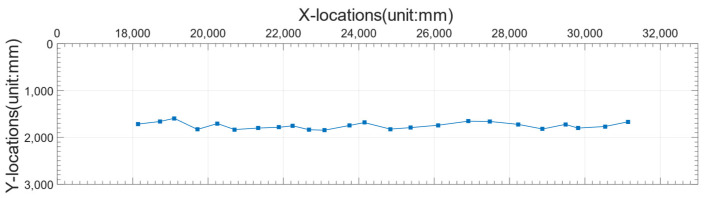
Detection of vehicles in field measurement.

Figure 15 shows the trajectory of the taxi on the upper-right corner in Figure 14b in the field measurement. The origin is the location of camera. The Y-axis is the transverse direction of the road, and the X-axis is the longitudinal direction of the road.

**Figure 16:**
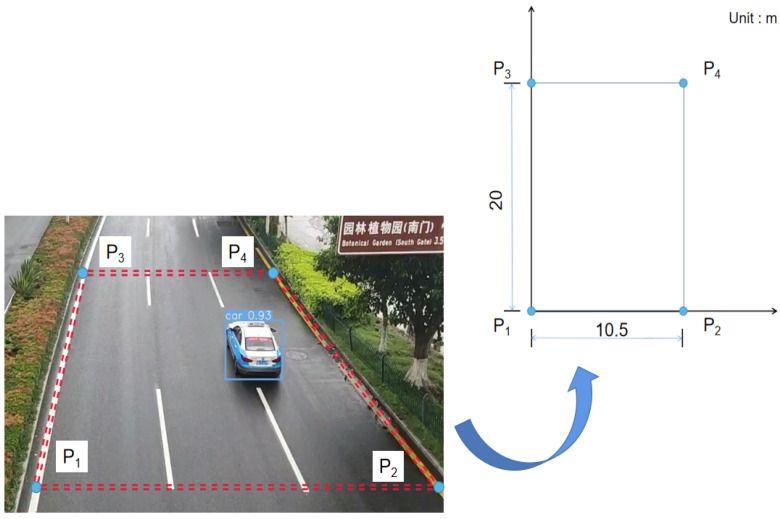
Reference points in the world coordinate system.

In order to verify the feasibility of the proposed method, the method requiring prior environment knowledge is used as a baseline or reference. Assuming that the road surface is flat, the specific relationship between coordinates in world coordinate system and pixel coordinate system can be written as

(10) $$Z_{c}\left[\begin{array}{c} u\\ v\\ 1 \end{array}\right]=\left[\begin{array}{c} \begin{array}{ccc} a_{11} & a_{12} & a_{13} \end{array}\\ \begin{array}{ccc} a_{21} & a_{22} & a_{23} \end{array}\\ \begin{array}{ccc} a_{31} & a_{32} & a_{33} \end{array} \end{array}\right]\left[\begin{array}{c} X_{w}\\ Y_{w}\\ 1 \end{array}\right]$$

where u and v are pixel coordinates, $X_{w}$ and $Y_{w}$ are world coordinates of points, the 3 × 3 matrix is the projection matrix, and $a_{33}$ is equal to 1. At least four pairs of reference points are required to determine the values of eight variables. As shown in Figure 16 and Table 3, one can obtain the pixel coordinates of P1, P2, P3 and P4 to calculate the projection transformation matrix.

**Figure 17:**
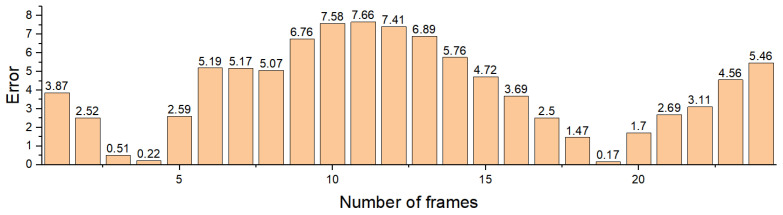
Distribution of error values.

Figure 17 shows the relative error of the proposed method compared to the baseline. It is found that the relative error is in the range from 0.17% to 7.66%, indicating that the proposed method using pose estimation to calculate the spatiotemporal information of passing vehicles also has great performance in field measurement, and also because prior environment knowledge is not required, which is convenient in real application.

## 7. Conclusions and Discussions

Compared with other work in this field, this study focused on identifying vehicle spatiotemporal information without prior spatial information of the road. Firstly, the deep learning-based vehicle recognition was introduced. YOLO-v5 was adopted in this study due to its unique engineering design. Through the training sets, the YOLO-v5 detector was used to identify the traffic flow, speed and other information, which is of great significance for understanding the traffic situation. Then, a pose estimation technology was proposed to solve the positioning problem of passing by vehicles. The traditional method can obtain accurate results when the vehicle is in the area where the prior environment knowledge is known, but it may fail once the vehicle is outside the area. The main advantage of the proposed method is to avoid the prior environment knowledge, and to use the camera itself as a reference point to estimate the vehicle location. This method can provide the spatial distribution of vehicles with respect to time and therefore it is expected to be applied to the traffic load estimation on bridges. The feasibility of this method was verified by lab-scale test and field measurement. The following findings and conclusions were drawn:

1. The proposed method can identify the spatiotemporal information of passing vehicles with high accuracy. Its accuracy is not dependent on the location of camera, so the camera can be installed at convenient locations.
2. There is no need to know the prior information of road before measurement, so marking on the road can be eliminated. Hence, it is possible to detect the spatiotemporal information of vehicles passing along curved path.
3. It is robust to camera disturbance and it can work as long as the camera is calibrated and the internal parameter matrix and distortion coefficient are known, so it is suitable for long-term monitoring.
4. It also has some limitations, such as high sensitivity to pixel coordinates. When the vehicle is far away, the estimation is less accurate. The results obtained by this method are also affected by poor environmental conditions such as extreme weak and strong light.
5. It is admitted that this method cannot fully automatically identify the spatiotemporal information of vehicles since it needs to select marked points manually. For the recognition principle and the effect of multiple vehicles in the same field of vision, there is little difference, but it will increase the workload. How to update the algorithm to fully automatically identify the spatiotemporal information of vehicles should be further investigated in the future.
6. The method of using binocular vision system to reduce the influence of pixels, and obtain more accurate vehicle marking points based on deep learning should also be explored, so as to obtain the spatiotemporal information of vehicle more conveniently. In addition, it is great to implement the algorithm model in embedded devices to achieve long-term monitoring.

## Figures and Tables

**Table 1:** Pixel coordinates of four selected points and identification results in Test 1.

A		B		C		D		X	Y	Error (x)
1142	1401	1484	1201	2450	2779	2821	2423	401.35	155.94	0.34%
975	1241	1323	1056	2163	2514	2531	2196	450.81	155.91	0.18%
818	1077	1165	903	1921	2251	2293	1961	502.68	155.45	0.54%
656	921	1004	752	1694	2004	2069	1739	550.43	155.11	0.08%
514	787	859	621	1471	1776	1857	1547	601.73	156.00	0.29%
397	647	730	499	1283	1576	1657	1357	648.08	155.65	0.30%
266	508	591	362	1101	1379	1454	1176	694.36	155.07	0.81%
144	389	469	253	927	1205	1273	1016	744.76	155.39	0.70%
42	274	361	139	776	1039	1120	871	791.45	155.36	1.07%

**Table 2:** Pixel coordinates of four selected points and identification results in Test 2.

A		B		C		D		X	Y	Error (x)
1862	1201	2201	1280	1225	2545	1659	2681	413.94	−186.97	3.49%
1931	1037	2269	1103	1352	2267	1766	2391	463.14	−194.12	2.92%
2010	878	2330	942	1475	2008	1873	2116	513.6	−193.21	2.72%
2077	726	2389	788	1587	1774	1967	1873	560.2	−188.45	1.85%
2145	583	2446	637	1688	1558	2056	1647	607.31	−193.19	1.22%
2206	448	2498	501	1780	1368	2133	1447	650.31	−194.18	0.05%
2257	325	2544	373	1860	1184	2202	1260	697.75	−190.90	0.32%
2312	206	2592	253	1941	1016	2270	1084	744.96	−191.64	0.67%
2363	96	2636	140	2018	855	2338	913	796.74	−193.05	0.41%

**Table 3:** Correspondence between two coordinate systems.

Points Number	*u* (Pixel)	*v* (Pixel)	*x* (m)	*y* (m)
P1	442	674	0	0
P2	1233	684	10.5	0
P3	534	280	0	20
P4	900	281	10.5	20

## Data Availability

Not applicable.
